# Introduction of the AmpliChip CYP450 Test to a South African cohort: a platform comparative prospective cohort study

**DOI:** 10.1186/1471-2350-14-20

**Published:** 2013-01-29

**Authors:** Tyren M Dodgen, Warren E Hochfeld, Heidi Fickl, Sahle M Asfaha, Chrisna Durandt, Paul Rheeder, Britt I Drögemöller, Galen E B Wright, Louise Warnich, Christiaan DJ Labuschagne, Antoinette van Schalkwyk, Andrea Gaedigk, Michael S Pepper

**Affiliations:** 1 Department of Pharmacology, University of Pretoria, Pretoria, South Africa; 2 Department of Immunology, University of Pretoria, Pretoria, South Africa; 3 Division of Clinical Epidemiology, School of Health Systems and Public Health, University of Pretoria, Pretoria, South Africa; 4 Department of Genetics, Stellenbosch University, Stellenbosch, South Africa; 5 Inqaba Biotechnical Industries, Pretoria, South Africa; 6 Section of Developmental Pharmacology and Experimental Therapeutics, Children’s Mercy Hospital & Clinics, Kansas City, Missouri, USA; 7 Department of Genetic Medicine and Development, University Medical Centre, University of Geneva, Geneva, Switzerland

## Abstract

**Background:**

Adverse drug reactions and lack of therapeutic efficacy associated with currently prescribed pharmacotherapeutics may be attributed, in part, to inter-individual variability in drug metabolism. Studies on the pharmacogenetics of Cytochrome P450 (CYP) enzymes offer insight into this variability. The objective of this study was to compare the AmpliChip CYP450 Test^®^ (AmpliChip) to alternative genotyping platforms for phenotype prediction of CYP2C19 and CYP2D6 in a representative cohort of the South African population.

**Methods:**

AmpliChip was used to screen for thirty-three *CYP2D6* and three *CYP2C19* alleles in two different cohorts. As a comparison cohort 2 was then genotyped using a *CYP2D6* specific long range PCR with sequencing (*CYP2D6* XL-PCR + Sequencing) platform and a PCR-RFLP platform for seven *CYP2C19* alleles.

**Results:**

Even though there was a low success rate for the AmpliChip, allele frequencies for both *CYP2D6* and *CYP2C19* were very similar between the two different cohorts. The *CYP2D6* XL-PCR + Sequencing platform detected CYP2D6*5 more reliably and could correctly distinguish between CYP2D6*2 and *41 in the Black African individuals. Alleles not covered by the AmpliChip were identified and four novel *CYP2D6* alleles were also detected*. CYP2C19* PCR-RFLP identified CYP2C19*9,*15, *17 and *27 in the Black African individuals, with *2, *17 and *27 being relatively frequent in the cohort. Eliminating mismatches and identifying additional alleles will contribute to improving phenotype prediction for both enzymes. Phenotype prediction differed between platforms for both genes.

**Conclusion:**

Comprehensive genotyping of *CYP2D6* and *CYP2C19* with the platforms used in this study, would be more appropriate than AmpliChip for phenotypic prediction in the South African population. Pharmacogenetically important novel alleles may remain undiscovered when using assays that are designed according to Caucasian specific variation, unless alternate strategies are utilised.

## Background

Inter-individual pharmacokinetic variability may account for the significant range in drug responses observed in the clinical setting. Response can be experienced both in terms of pronounced adverse drug reactions (ADRs) and inability to reach therapeutic levels. Cytochrome P450 (CYP) enzymes are estimated to be responsible for up to 86% of Phase I metabolism of commonly prescribed therapeutic drugs [1]. Of the CYP enzymes, CYP2D6 and CYP2C19 have been estimated to metabolise approximately 25% [2] and 8% [3] of these commonly prescribed drugs, respectively. CYP2D6 is involved in the metabolism of antidepressants, selective serotonin reuptake inhibitors, antipsychotics antiarrhythmics, *β*-blockers and opioid analgesics; while CYP2C19 is involved in the metabolism of proton pump inhibitors, benzodiazepines, tricyclic antidepressants, selective serotonin reuptake inhibitors, barbiturates, anti-malarial agents, anticonvulsants, monoamine oxidise inhibitors and platelet aggregation inhibitors [4-6].

In an effort to explain pharmacokinetic variability, genetic mutations present in drug metabolising enzymes have been the predominant focus of pharmacogenetic studies. Due to the complexity and vast number of mutations present in these genes, the Human Cytochrome P450 (*CYP*) Allele Nomenclature website was created in order to catalogue genetic variability in CYP enzymes (http://www.cypalleles.ki.se/). Over 100 alleles for *CYP2D6* and 28 alleles for *CYP2C19* have been described to date (27 November 2012). For a subset of the alleles, *in vivo* and/or *in vitro* studies have elucidated enzyme activities and these activities are listed as increased, normal, decreased or none. This information can be used, along with genotype, to predict the poor (PM), intermediate (IM), extensive (EM) or ultra-rapid metaboliser (UM) status of the *CYP* genes [6]. Clinicians could potentially use this predicted metaboliser status to personalise prescription, with the intention of reducing ADRs and increasing therapeutic efficacy. Pharmacogenetics has been estimated to potentially reduce ADRs by 10-20% and to improve efficacy by 10-15%, and underlies the rationale for pharmacogenetic screening [5].

In order for a pharmacogenetic screening assay to be effective, it must be able to deal with highly polymorphic genes with high throughput capability in an efficient and cost effective way. The Roche AmpliChip CYP450 Test^®^ (AmpliChip) was created with this in mind. In 2005, this Affymetrix platform (Roche Molecular Systems, Inc., Branchburg, NJ) became the first DNA based microarray to be approved by the Food and Drug Administration (FDA) for *CYP2C19* and *CYP2D6* pharmacogenetics [7]. The AmpliChip is a high-throughput, comprehensive screening assay designed to simultaneously identify thirty-three *CYP2D6* and three *CYP2C19* alleles from whole blood-derived DNA (http://www.amplichip.us/documents/CYP450_P.I._US-IVD.pdf). In an initial assessment of the AmpliChip, de Leon et al. [8] said that, “this new technology is a major step in ushering ‘personalized prescription’ into the clinical environment.” Rebsamen et al. [9] observed that the AmpliChip is good at predicting PMs and EMs, satisfactory in predicting IMs, but not as efficient at predicting UMs. In summarising, Rebsamen et al. [9] stated that, “this microarray technology could be an excellent tool to improve phenotype prediction.” The AmpliChip has been validated for *CYP2D6* on German Caucasians (n=158, [10]), female Swiss Caucasians (n=165, [9]) and a combined Caucasian (n=3779) and African American (n = 452) cohort [7]. Heller et al. [10] concluded that the AmpliChip was fast, accurate and comprehensive in its identification of *CYP2D6* genotype and predicted phenotype. A summary of these articles can be found in Table 1 where notably it appears that there are more PMs in Caucasians than in Black Africans and Koreans [7,9-13]. The only group to report results for *CYP2C19* was de Leon et al. [7]. This study found that 98.0% of American Caucasians were EM and 2.0% were PM (cohort: n=3938), with and allele frequency of 14.2% for CYP2C19*2 and 0.0% for *3. In comparison 96.0% of African Americans (cohort size=478) were predicted to be EM and 4% were predicted to be PM, with allele frequencies of 18.3% for CYP2C19*2 and 0.1% for *3[7].

**Table 1:** **Summary of reported *CYP2D6 *genotyping studies using the AmpliChip CYP450 test**

**Article**	**Nikoloff et al. 2002**		**Ishida et al. 2002**	**Heller et al. 2006**	**Rebsamen et al. 2009**	**de Leon et al. 2009**		**Ramόn y Cajal et al. 2010**
**Cohort**	**TD+ Schizophrenic Koreans**	**TD- Schizophrenic Koreans**	**Japanese**	**German Caucasians**	**Swiss female Caucasians**	**American Caucasians**	**African Americans**	**Tamoxifen treated Spanish Caucasians**
**Variant**	**Allele frequency (%)**							
*1	38.2	40.7	46.9	36.2	35.5	37.4	59.7	35.7
*2	9.1	9.2	19.8	10.5	15.5	15.9	6.0	16.5
*3	0.0	0.0	0.0	3.0	0.6	1.8	0.2	0.5
*4	0.0	0.0	0.0	12.2	20.6	21.0	5.5	14.8
*5	3.6*	1.6*	-	8.2	2.4	2.3	2.8	4.9
*6	0.0	0.0	0.0	2.6	1.2	1.1	0.2	0.5
*7	0.0	0.0	0.0	0.0	0.3	0.0	0.0	0.0
*8	0.0	0.0	0.0	0.0	0.0	0.0	0.0	0.0
*9	0.0	0.0	0.0	0.7	2.7	2.9	0.4	7.1
*10	47.3	46.1	33.3	1.6	2.7	1.0	3.8	4.4
*11	0.0	0.0	0.0	0.0	0.0	0.0	0.0	0.0
*14	0.5	0.0	0.0	0.0	0.0	0.0	0.0	0.0
*15	-	-	-	0.0	0.0	0.0	0.0	0.0
*17	-	-	-	0.0	0.3	0.3	18.4	0.0
*18	0.0	0.0	0.0	0.0	0.0	0.0	0.0	0.0
*19	0.0	0.0	0.0	0.0	0.0	0.0	0.0	0.0
*20	-	-	-	0.0	0.0	0.0	0.0	0.5
*25	0.0	0.0	0.0	0.0	0.0	0.0	0.0	0.0
*26	0.0	0.0	0.0	0.0	0.0	0.0	0.0	0.0
*29	-	-	-	0.0	0.3	0.2	7.7	0.0
*30	-	-	-	0.0	0.0	0.0	0.0	0.0
*31	0.0	0.0	0.0	0.0	0.0	0.0	0.0	0.0
*35	-	-	-	6.6	7.3	4.8	0.9	4.4
*36	0.0*	0.0*	-	0.0	0.0	0.0	0.6	0.0
*40	-	-	-	0.0	0.0	0.0	0.3	0.0
*41	1.4	2.2	0.0	6.3	7.3	9.8	14.9	9.3
*1xn	-	-	-	6.6	0.6	0.7	0.8	0.5
*2xn	-	-	-	4.9	1.5	0.5	1.2	0.5
*4xn	-	-	-	0.3	0.3	0.1	2.4	0.0
*6xn	-	-	-	0.0	0.0	0.0	0.0	0.0
*10xN	-	-	-	0.0	0.0	0.0	0.0	0.0
*17xN	-	-	-	0.0	0.0	0.0	0.2	0.0
*29xN	-	-	-	0.0	0.0	0.0	0.2	0.0
*35xN	-	-	-	0.3	0.0	0.0	0.0	0.0
*41xN	-	-	-	0.0	0.9	0.1	0.0	0.0
**Total alleles**	220	184	162	304	330	7558	904	182
**Predicted phenotype**								
**PM**	0 (0.0)	0 (0.0)	Not reported	10.5 (16)	9.1 (15)	8.2 (311)	1.8 (8)	6.6 (6)
**IM**	23.6 (26)	25 (23.0)		4.0 (6)	7.9 (13)	9.7 (365)	32.7 (148)	11.0 (10)
**EM**	76.4 (84)	75 (69.0)		71.7 (109)	81.8 (135)	80.7 (3048)	63.5 (287)	81.3 (74)
**UM**	-	-		13.8 (21)	1.2 (2)	1.5 (55)	2.0 (9)	1.1 (1)
**Cohort**	110	92	81	152	165	3779	452	91

Alleles not covered by AmpliChip at the time of reporting are represented by “—”. The microarray used in the Nikoloff et al. (2002) paper did not cover CYP2D6*5; however, a separate assay was used for *5 detection and the frequency was reported. Predicted phenotype reported or adjusted to represent AmpliChip test calls. TD+; patients with tardive dyskinesia, TD- patients without tardive dyskinesia.

Although several populations of European descent have been investigated using the AmpliChip, this assay has not been used to genotype an African population residing in Africa. Considering that novel alleles have been found in African cohorts [14-18], it is important to evaluate these genetically diverse populations when considering pharmacogenetic implementation. This needs to be addressed, given that ADRs occur in an estimated 14% of hospitalised South African patients resulting in a 5–10 fold higher fatality compared to USA and UK hospitals [19]. The implementation of a pharmacogenetic assay may assist in reducing the socio-economic burden associated with this sub-optimal treatment in South Africa. The objective of this study was therefore to evaluate the AmpliChip for use as a pharmacogenetic screening tool for *CYP2D6* and *CYP2C19* in the South African population.

## Methods

### Study subjects and sampling

Ethical approval was obtained from the Research Ethics Committee, Faculty of Health Science, University of Pretoria (Approval numbers: Cohort 1 - 102/2005 and Cohort 2 - S132/2009) and the study was conducted in accordance with the Declaration of Helsinki, using GCP guidelines. All participating volunteers were ≥18 years of age, South African citizens and resided in the city of Pretoria during the sampling period. These cohorts were chosen to be demographically representative of the general population of South Africa (http://www.statssa.gov.za/). It should be noted however, that it is not the authors’ intention to use this study for inter-ethnic comparisons. Informed consent was obtained from all participants along with general demographic information including place of birth and voluntary disclosure of ethnic group (Black African, Caucasian, Coloured and Indian). The term Coloured, also referred to as Mixed Ancestry in the South African context, is used officially to describe an admixed group of people predominantly residing in the Western Cape [20,21]. The admixture present in this population is derived from several diffe rent ancestries including European, Asian and African, primarily Khoisan and Bantu influence. The high level of admixture can be attributed to the presence of the major trade routes in South Africa during the fifteenth to nineteenth centuries [20,21].

#### Cohort 1

Diabetic individuals (n=83): 57 Black African; 6 Caucasian; 10 Coloured and 10 Indian. These individuals were attending the Diabetic Clinic at the Steve Biko Academic Hospital in Pretoria. This cohort was genotyped using AmpliChip.

#### Cohort 2

Apparently healthy volunteers (n=100) were recruited from several different sites in Pretoria. This cohort consisted of 70 Black African, 10 Caucasian, 10 Coloured and 10 Indian individuals. This cohort was used to comparatively evaluate the AmpliChip platform using PCR-RFLP and XL-PCR+Sequencing for *CYP2C19* and *CYP2D6* respectively.

### Genomic DNA (gDNA) extraction

Venous blood samples collected in ethylenediaminetetraacetic acid (EDTA) vacutainer tubes (Becton-Dickinson, Franklin Lakes, NJ, USA) were used for gDNA extraction. Extraction was performed using the Genomic DNA Purification Kit (Fermentas Life Science, Lithuania) or the automated Maxwell^®^ 16 system (Promega, Madison, WI, USA), and extraction was performed according to the manufacturer’s instructions.

### AmpliChip CYP450 Test

Each sample was simultaneously evaluated for *CYP2D6* and *CYP2C19* using the AmpliChip CYP450 Test (Roche Molecular Systems Inc., Pleasanton, CA, USA) according to the manufacturer’s protocol (AmpliChip CYP450 Test package insert). In brief, *CYP2D6* and *CYP2C19* were amplified in two separate reactions. Both reactions were monitored for amplification using 1.0% agarose gel electrophoresis for 1 hour (not in protocol). Reactions were pooled and subjected to *DNase* I (Roche Molecular Systems Inc.) fragmentation, following which the fragments were 3’-end labelled using Terminal Transferase (Roche Molecular Systems Inc.) and TdT Labelling Reagent (supplied in the AmpliChip kit).

Using a pre-programmed protocol, the labelled fragments were hybridised onto AmpliChip CYP450 microarrays, stained with streptavidin-conjugated phycoerythrin (Invitrogen Corp., Carlsbad, CA, USA) and washed in an Affymetrix GeneChip Fluidics Station 450Dx (Affymetrix, Santa Clara, CA, USA). Each AmpliChip was then scanned with an Affymetrix GeneChip Scanner 3000Dx (Affymetrix). The resulting image was orientated using GeneChip Operating Software (Affymetrix) and transferred to AmpliChip CYP450 Data Analysis Software (Roche Molecular Systems Inc.) to determine *CYP2D6* and *CYP2C19* genotypes and predicted phenotypes.

### *CYP2C19* Genotyping

The same gDNA samples analysed by AmpliChip were also analysed by a PCR-RFLP platform designed for South African Xhosa individuals [18]. This assay was used to evaluate the ability of AmpliChip to genotype the *CYP2C19* variation present in the South African population. Alleles identified and assayed were named according the *CYP* Allele Nomenclature Committee’s online database (http://www.cypalleles.ki.se/). This platform focuses on identifying allele defining SNPs for CYP2C19*2, *3, *9, *15, *17, *27 and *28 alleles (method summarised in Additional file 1: Table S1 and Additional file 2: Table S2).

### *CYP2D6* Long range PCR with sequencing

A *CYP2D6* Long Range PCR with Sequencing (XL-PCR+Sequencing) strategy was designed to genotype Cohort 2 and to assess the ability of AmpliChip to successfully genotype the functionally significant alleles present in the South African population. This alternate approach included a series of long range PCR (XL-PCR) amplifications which were used for detection of CYP2D6*5 (complete gene deletion), *CYP2D6* duplication (increased copy number) and to amplify a *CYP2D6* product for sequencing (introns and exons). All primers (Additional file 3: Table S3 and Additional file 4: Table S4) utilised for amplification were manufactured by Inqaba Biotechnical Industries (Pretoria, South Africa). The amplification reactions (in detail below) were performed using a Gold-plated 96-Well GeneAmp 9700 thermal cycler (Applied Biosystems, Foster City, CA, USA), followed by electrophoresis using 1.0% agarose gels for 1 hour.

#### XL-PCR reactions detection of CYP2D6*5 and duplications

CYP2D6*5 detection was based on a duplex XL-PCR assay described by Hersberger et al. [22]. This reaction was performed using Long-Range Taq polymerase (Fermentas Life Science). PCR reaction conditions were optimised for primer concentration and denaturing time to ensure equal amplification of the CYP2D6*5 deletion fragment (3.2 kb) and the whole *CYP2D6* gene fragment (5.1 kb)*.* Heterozygous samples were repeated using only the *CYP2D6* specific primers in order to generate the 5.1 kb amplicon for sequencing.

The XL-PCR duplex amplification reaction described by Gaedigk et al. [23] was used to detect the presence of *CYP2D6* duplications (primers and conditions in Additional file 3: Table S3). A separate XL-PCR reaction amplified a duplication-specific product allowing amplification and characterisation of allelic status of the duplicated gene [23]. The duplication-specific product was characterised by re-sequencing (primers and conditions in Additional file 3: Table S3).

#### CYP2D6 re-sequencing

Prior to re-sequencing, amplified PCR products were purified using Exonuclease I and FastAP™ Thermosensitive Alkaline Phosphatase (Fermentas Life Science) [24]. Sanger sequencing was done by Inqaba Biotechnological Industries using the ABI Big Dye Terminator Cycle Sequencing kit version 3.1 and 3130 XL and 3500XL sequencer systems (Applied Biosystems Inc.) and primers described in Additional file 4: Table S4.

Electropherograms were edited using FinchTV version 1.4.0 (Copyright © 2004–2006, Geospiza Inc.). Following editing, sequences were imported into CLC DNA Workbench version 5.5 (CLCBio, Aarhus, Denmark), assembled and compared to the *CYP2D6* reference sequence AY545216 (GenBank). As with the AmpliChip, *CYP2D6* sequence variations were numbered and alleles were assigned according the P450 Nomenclature Committee website.

#### Evaluation of exon 9 gene conversion

The presence of non-functional CYP2D6*4 N and *36 allelic variants where evaluated by assaying for the presence of a *CYP2D7* gene conversion in exon 9. The PCR reaction (primers and conditions in Additional file 3 Table S3) was performed as described by Gaedigk et al. [25] using BIOTAQ^™^ DNA Polymerase (Bioline, London, UK). The amplicon was analysed using 3% agarose gel electrophoresis.

#### Characterisation of novel alleles

To characterise haplotypes associated with novel non-synonymous SNPs, a 6.6 kb long PCR product was amplified using *CYP2D6* specific primers described previously [23]. This product was cloned using the CloneJET^™^ PCR Cloning Kit (Fermentas Life Science) according to manufacturer’s instructions and transformed into DH5α cells (Zymo Research, Orange, CA, USA). Colonies were screened by amplifying the region of interest (where the novel SNP was located) using relevant sequencing primers followed by sequencing. Once the correct colony was identified, colony extraction was performed using Zuppy^™^ Plasmid Miniprep Kit (Zymo Research) and sequenced as described above. The haplotype of the novel allele was determined by comparing the sequence obtained from the cloned allele and the sequence of the XL-PCR product representing both alleles. Novel allele defining non-synonymous SNPs were analysed using “sorting intolerant from tolerant” (SIFT) and PolyPhen prediction software which estimates the effect on CYP2D6 activity *in silico*[26,27]. Potential splice site variation was evaluated *in silico* using NetGene2 [28,29]. Novel allele sequences were submitted to the *CYP* Allele Nomenclature Committee for *CYP2D6* allele designation.

### Phenotype prediction

AmpliChip software predicted phenotype based on principles explained in Table 2 (AmpliChip CYP450 Test package insert). The Activity Score (AS) model [30] was used to predict phenotype from data generated by *CYP2D6* re-sequencing and the AmpliChip. AS was calculated using model A [30]. Novel alleles were assigned an AS of 1.0 to allow for phenotypic comparison, since actual enzyme activity has not yet been confirmed. The exception was CYP2D6*4P; its novel non-synonymous SNP was linked with 1846 G>A, the CYP2D6*4-defining SNP that causes a splice defect thereby obliterating activity (AS=0). The AS was also adopted to predict CYP2C19 phenotype, which is explained in Table 2.

**Table 2:** CYP2D6 and CYP2C19 phenotype prediction

***Estimated metabolic potential of alleles***			
***CYP2D6***	**Allele activity**	**Numeric activity**	***CYP2C19***
*1xN, *2xN	Increased	2.0	*17
*1,*2, *22, *33, *35, *43, *45B, *46	Normal	1.0	*1+, *28
*10, *17, *29, *41, *59	Decreased	0.5	*9, *27
*4, *5, *14, *16, *40, *56B, *4xN	Absent	0.0	*2, *3
*25, *30, *64, *65, *73, *74, *84, *85, *86	Unknown	1.0	*15
(Activity according to http://www.cypalleles.ki.se/)			
***Phenotype prediction***			
**AmpliChip**	**Prediction**		**Activity Score (AS)**
3 or more functional alleles	UM		> 2.0
1 or 2 functional alleles and increased paired with decreased or absent	EM		1.5-2.0
1 or 2 reduced function alleles	IM		0.5-1.0
2 absent function alleles	PM		0.0

Alleles for both genes present in this table are relevant to this study; additional allele information is available at http://www.cypalleles.ki.se/. The activity of each allele in the genotype is used to predict phenotype. CYP2D6 Activity Score (AS) is assigned according to model A proposed by Gaedigk et al. (2008). The AS was adopted to predict CYP2C19 activity.

### Statistics

Tools for Population Genetic Analysis (TFPGA) software v1.3 (Miller, 1997: http://www.marksgeneticsoftware.net/tfpga.htm) was used (i) to test allele deviation from a Hardy-Weinberg equilibrium using a Fisher’s exact test for each ethnic group within each cohort and (ii) for comparing platforms using Fisher’s exact test. Linkage disequilibrium was evaluated using Haploview software v3.31 [31]. A *P* value of <0.05 was considered to be significant.

## Results

### Success rate of the AmpliChip CYP450 Test

Cohort 1 (n=83) had a success rate with AmpliChip of 75.9% for *CYP2D6* and 98.8% for *CYP2C19*. There were five “No Calls” (all hybridisation positions were occupied and identified by the AmpliChip software, but based on the hybridisation pattern a genotype could not be generated, nor a phenotype predicted) for *CYP2D6*, raising the success rate of the AmpliChip to 81.9% with only 75.9% generating pharmacogenetically relevant data. None of the failed AmpliChips were repeated for this group.

Cohort 2 (n=100) had a success rate of 71.0% for *CYP2D6*. Of the AmpliChip micorarrays which failed to generate a genotype, 4.0% were “No Calls”. Therefore, 75.0% of the microarrays were successful, of which only 71.0% gave pharmacogenetically relevant results. The most frequent hybridisation failures in both cohorts were at the 1758 G locus, which is associated with CYP2D6*8 (1758 G>T) and *14 (1758 G>A) alleles. The AmpliChip information leaflet mentioned that this would indeed be the most likely hybridisation locus to fail. For *CYP2C19*, 100.0% of the AmpliChips generated a genotype, and a predicted phenotype could thus be assigned in all cases.

Thirteen failed samples and two successful samples (positive controls) were repeated in order to estimate user error. The two samples which had succeeded previously were again successful. Of the thirteen failures, two succeeded, three failed (but hybridised at additional loci), one failed at different loci and the balance failed as they did before (missing the same hybridisation loci).

### *CYP2C19* genotype analysis

#### AmpliChip

Using AmpliChip to evaluate genotype, it was found that there were no statistically significant differences in *CYP2C19* allele frequencies between the two sampled cohorts (*P*>0.08) and all alleles were in Hardy-Weinberg equilibrium in both cohorts (Table 3). Typically rare, CYP2C19*3 only occurred in Cohort 1, but was relatively infrequent and not statistically significant.

**Table 3:** ***CYP2C19 *allele and predicted phenotype frequency in a demographically representative South African (SA) cohort (n=100) compared to other cohorts sampled in SA**

		**Cohort 1 AmpliChip**				**Comparison**	**Cohort 2 AmpliChip**				**Comparison**	**PCR-RFLP**				**Dandara et al. 2001**	**Dandara et al. 2011 **[32]	**Drögemöller et al. 2010**		**Ikediobi et al. 2011 **[33]		**Matimba et al. 2009**
**Allele**	**Activity**	**Black African**	**White Caucasian**	**Coloured**	**Indian**	**Fischer's Exact**	**Black African**	**White Caucasian**	**Coloured**	**Indian**	**Fischer's Exact**	**Black African**	**White Caucasian**	**Coloured**	**Indian**	**SA Venda**	**SA Bt20 (mixed Black)**	**SA Xhosa**	**SA Coloured**	**SA Xhosa**	**SA Coloured**	**SA Venda**
**Allele Frequency (%)**																						
*1+	Normal	85.7	75.0	75.0	60.0	1.000	82.1	95.0	65.0	65.0	0.000	32.1	70.0	40.0	40.0	78.0	23.0	17.0	41.0	63.0	71.0	77.0
*2	Absent	116	25.0	25.0	40.0	0.784	17.9	5.0	35.0	35.0	1.000	17.9	5.0	35.0	35.0	22.0	61.0	21.0	17.0	22.0	20.0	17.0
*3	Absent	2.7	0.0	0.0	0.0	0.084	0.0	0.0	0.0	0.0	1.000	0.0	0.0	0.0	0.0	0.0	16.0	0.0	7.0	-	-	0.0
*9	Decreased	-	-	-	-	-	-	-	-	-	0.058	3.6	0.0	0.0	0.0	-	-	9.0	4.0	-	-	6.0
*15	Unknown	-	-	-	-	-	-	-	-	-	0.002	5.7	0.0	10.0	0.0	-	-	9.0	8.0	-	-	0.0
*17	Increaased	-	-	-	-	-	-	-	-	-	0.000	16.4	25.0	0.0	5.0	-	-	10.0	14.0	15.0	9.0	-
*27	Decreased	-	-	-	-	-		-	--	-	0.000	24.3	0.0	15.0	20.0	-	-	33.0	8.0	-	-	-
*28	Unknown	-	-	-	-	-	-	-	-	-	1.000	0.0	0.0	0.0	0.0	-	-	1.0	1.0	-	-	0.0
**Alleles identified (n)**		112.0	12.0	20.0	20.0		140.0	20.0	20.0	20.0		140.0	20.0	20.0	20.0	152.0	1964-1970	200.0	150.0	218.0	134.0	18.0
**Predicted Phenotype Frequency (%)**																						
**PM**		3.5	16.7	10.0	30.0		7.1	0.0	10.0	10.0		5.7	0.0	20.0	50.0	5.3	1.5	3.0	8.0	-	-	-
**IM**		0.0	0.0	0.0	0.0		0.0	0.0	0.0	0.0		21.4	10.0	50.0	40.0	32.9	13.0	49.0	40.0	-	-	-
**EM**		94.7	83.3	90.0	70.0		92.9	100.0	90.0	90.0		45.7	90.0	30.0	0.0	61.6	85.5	39.0	35.0	-	-	-
**UM**		0.0	0.0	0.0	0.0		0.0	0.0	0.0	0.0		27.1	0.0	0.0	10.0	0.0	0.0	9.0	17.0	-	-	-
**Failure**		1.8	0.0	0.0	0.0		0.0	0.0	0.0	0.0		0.0	0.0	0.0	0.0	0.0	1.1-0.8	0.0	0.0	0.0	0.0	0.0
**Cohort (n)**		57	6	10	10		70	10	10	10		70	10	10	10	76	982-985	100	75	109	67	9

Allele frequencies for each allele were compared between cohorts and platforms. Allele frequencies for different ethnicities were summed for comparison. Genetic material for cohort 2 was genotyped using PCR-RFLP to evaluate AmpliChip CYP450 Test^®^ (AmpliChip) genotype calls. CYP2C19*1+ : wild type and any unidentified alleles; —:alleles not identified by assay or predicted phenotype not reported.

#### PCR-RFLP

The PCR-RFLP platform identified high frequencies (refer to Table 3; *P*≤0.002) of CYP2C19*15 (unknown), *17 (increased metabolism) and *27 (decreased metabolism). Although not significant (*P=*0.058) when combining ethnicities, CYP2C19*9 (decreased metabolism) was present at high frequency (*P=*0.029) over the whole cohort, when only Black Africans were compared between platforms. Interestingly, four samples (three Black Africans and one Indian) were homozygous for CYP2C19*2, but were also heterozygous for the *27 allele. This suggests that the 19154 G>A and -1401 G>A SNPs used for CYP2C19*2 and *27 detection respectively, may be in partial LD with one another, forming an additional allele. The combination was listed as CYP2C19*2, since the presence of the 19154 G>A splicing defect would be the allele-defining SNP as it causes a non-functional gene product.

#### Predicted Phenotype

The only difference between the two cohorts for AmpliChip predicted phenotype was PM for White Caucasians, as there were more identified in cohort 1. Caution should be taken when making this comparison, as this 16.7% frequency is only one individual in the cohort and the sample size is not statistically large enough to make a valid comparison. The adoption of AS combined with *CYP2C19* PCR-RFLP allows IMs to be assigned and also changes the identification profile of PMs (refer to Table 3). EM and PM predicted phenotype in Black Africans following *CYP2C19* PCR-RFLP correlated well with the Xhosa individuals screened by Drögemöller et al. [18], but IM and UM seem to be different.

### *CYP2D6* genotype analysis

#### AmpliChip

Table 4 summarises the *CYP2D6* allele frequencies for the sampled cohorts and compares allele frequencies between cohorts and platforms [14,17,34]. The only allele which was out of Hardy-Weinberg equilibrium was CYP2C19*10 (*P*=0.006) in cohort 1. CYP2D6*17 was the only allele with significant allele frequency differences (*P*=0.045) between the two cohorts which is likely to be the result of a larger number of Black Africans in cohort 2. AmpliChip found Black African individuals in both cohorts to have a relatively high frequency of CYP2D6*17 and CYP2D6*41. CYP2D6 *4 and *41 were relatively frequent in the Caucasian, Coloured and Indian populations in both cohorts and could be a source for potential PM (refer to Table 4).

**Table 4:** ***CYP2D6 *allele and predicted phenotype frequency in a demographically representative South African (SA) cohort (n=100) compared to other cohorts sampled in SA**

		**Cohort 1 AmpliChip**				**Comparison**	**Cohort 2 AmpliChip**				**Comparison**	**XL-PCR + Sequencing**				**Dandara et al. 2001**	**Gaedigk et al. 2008**	**Wright et al. 2010**	
**Allele**	**Activity**	**Black African**	**White Caucasian**	**Coloured**	**Indian**	**Fischer's Exact**	**Black African**	**White Caucasian**	**Coloured**	**Indian**	**Fischer's Exact**	**Black African**	**White Caucasian**	**Coloured**	**Indian**	**SA Venda**	**SA Coloureds**	**SA Xhosa Control**	**SA Xhosa Schizophrenia**
**Allele Frequency (%)**																			
*1	Normal	30.7	40.0	38.9	30.0	0.301	20.0	31.0	37.5	60.0	0.715	25.7	30.0	30.0	45.0	50.0	26.8	23.6	24.5
*2	Normal	4.5	20.0	0.0	30.0	1.000	20.	18.8	18.8	30.0	0.107	8.6	15.0	15.0	30.0	17.8	15.2	12.3	15.7
*4	Absent	2.3	10.0	16.7	10.0	0.795	0.0	31.3	12.5	0.0	0.586	0.0	20.0	15.0	0.0	3.3	7.1	1.9	1.0
*5	Absent	5.7	10.0	0.0	0.0	0.523	4.0	0.0	0.0	0.0	0.037	10.7	5.0	5.0	0.0	4.6	17.2	14.2	18.6
*10	Decreased	6.8	0.0	0.0	10.0	1.000	6.0	6.3	6.3	0.0	0.808	5.7	5.0	5.0	0.0	-	2.5	1.9	2.0
*14	Absent	0.0	0.0	0.0	0.0	1.000	0.0	0.0	0.0	0.0	1.000	0.0	0.0	0.0	0.0	-	0.5	0.0	0.0
*16	Absent	-	-	-	-	-	-	-	-	-	1.000	0.0	0.0	0.0	0.0	-	0.5	0.0	0.0
*17	Decreased	13.6	0.0	11.1	0.0	0.045	31.0	0.0	0.0	0.0	0.584	25.7	0.0	10.0	0.0	24.0	12.6	13.2	16.7
*22	Normal	-	-	-	-	-	-	-	-	-	1.000	0.0	0.0	0.0	5.0	-	—	—	—
*25	Unknown	0.0	0.0	0.0	0.0	1.000	1.0	0.0	0.0	0.0	0.416	0.0	0.0	0.0	0.0	-	—	—	—
*29	Decreased	13.6	0.0	0.0	0.0	1.000	6.0	0.0	6.3	0.0	0.598	4.3	0.0	5.0	0.0	-	4.6	13.2	6.9
*30	Unknown	1.1	0.0	0.0	0.0	0.461	0.0	0.0	0.0	0.0	1.000	0.0	0.0	0.0	0.0	-	—	0.0	0.0
*33	Normal	-	-	-	-	-	-	-	-	-	1.000	0.0	5.0	0.0	0.0	-	—	—	—
*35	Normal	0.0	0.0	0.0	0.0	1.000	0.0	0.0	6.3	0.0	1.000	0.0	5.0	0.0	0.0	-	—	—	—
*36	Reduced	0.0	0.0	0.0	0.0	1.000	0.0	0.0	0.0	0.0	1.000	0.0	0.0	0.0	0.0	-	0.0	0.0	0.0
*40	Absent	1.1	10.0	5.6	0.0	1.000	3.0	0.0	0.0	0.0	1.000	3.6	0.0	0.0	0.0	-	0.0	1.9	2.9
*41	Decreased	20.5	10.0	27.8	20.0	0.883	26.0	12.5	12.5	10.0	0.000	0.7	15.0	5.0	10.	-	0.0	1.9	1.0
*43	Normal	-	-	-	-	-	-	-	-	-	0.272	0.7	0.0	5.0	5.0	-	—	0.9	1.0
*45B	Normal	-	-	-	-	-	-	-	-	-	0.020	5.7	0.0	0.0	0.0	-	0.0	10.4	1.0
*46	Normal	-	-	-	-	-	-	-	-	-	1.000	0.7	0.0	0.0	0.0	-	0.0	0.0	0.0
*56B	Absent	-	-	-	-	-	-	-	-	-	1.000	0.7	0.0	0.0	0.0	-	0.0	—	—
*59	Decreased	-	-	-	-	-	-	-	-	-	1.000	0.0	0.0	0.5	0.0	-	—	—	—
*64	Unknown	-	-	-	-	-	-	-	-	-	1.000	0.0	0.0	0.0	0.0	-	0.0	0.0	0.0
*65	Unknown	-	-	-	-	-	-	-	-	-	1.000	0.0	0.0	0.0	0.0	-	0.0	0.0	0.0
*73	Unknown	-	-	-	-	-	-	-	-	-	1.000	0.0	0.0	0.0	0.0	-	—	0.0	1.0
*74	Unknown	-	-	-	-	-	-	-	-	-	1.000	0.0	0.0	0.0	0.0	-	—	0.0	1.0
*84	Normal	-	-	-	-	-	-	-	-	-	1.000	0.7	0.0	0.0	0.0	-	—	—	—
*85	Normal	-	-	-	-	-	-	-	-	-	1.000	0.7	0.0	0.0	0.0	-	—	—	—
*86	Normal	-	-	-	-	-	-	-	-	-	1.000	0.0	0.0	0.0	5.0	-	—	—	—
*1xN	Increased	0.0	0.0	0.0	0.0	1.000	0.0	0.0	0.0	0.0	1.000	0.0	0.0	0.0	0.0	-	0.0	0.0	1.0
*2xN	Increased	0.0	0.0	0.0	0.0	1.000	1.0	0.0	0.0	0.0	1.000	0.7	0.0	0.0	0.0	-	0.0	2.8	2.9
*4xN	Absent	0.0	0.0	0.0	0.0	1.000	0.0	0.0	0.0	0.0	0.147	2.9	0.0	0.0	0.0	-	0.0	1.9	2.9
Hybrid alleles		-	-	-	-	-	-	-	-	-	-	-	-	-	-	-	0.0	0.0	0.0
**Alleles identified (n)**		88	10	18	10		100	16	16	10		140	20	20	20	152	198	106	102
**Predicted Phenotype Frequency (%)**																			
PM		3.5	83.3	10.0	0.0		0.0	10.0	0.0	0.0		0.0	10.0	0.0	0.0	2.6	3.0	3.8	7.8
IM		28.1	0.0	20.0	0.0		42.9	10.0	20.0	0.0		42.9	30.0	50.0	0.0	56.6	53.0	47.2	37.3
EM		43.9	0.0	60.0	50.0		27.1	60.0	60.0	50.0		57.1	60.0	50.0	100.0	40.8	39.0	43.4	47.1
UM		0.0	0.0	0.0	0.0		0.0	0.0	0.0	0.0		0.0	0.0	0.0	0.0	0.0	4.0	5.7	7.8
Unknown		1.8	0.0	0.0	0.0		1.4	0.0	0.0	0.0		0.0	0.0	0.0	0.0	0.0	0.0	0.0	0.0
No call		7.0	0.0	0.0	0.0		5.7	0.0	0.0	0.0		0.0	0.0	0.0	0.0	0.0	0.0	0.0	0.0
Failure		15.8	16.7	10.0	40.0		22.9	20.0	20.0	50.0		0.0	0.0	0.0	0.0	0.0	0.0	0.0	0.0
Cohort (n)		57	6	10	10		70	10	10	10		70	10	10	10	76	99	53	51

Allele frequencies for each allele were compared between cohorts and platforms for statistical comparison. Allele frequencies for different ethnicities were summed for comparison. Genetic material for cohort 2 was sequenced to evaluate AmpliChip CYP450 Test^®^ (AmpliChip) genotype calls. Lower allele numbers were reported for the AmpliChip due to “No Calls” and failed chips. Individuals could be predicted as poor (PM), intermediate (IM), extensive (EM) and ultrarapid metabolisers using AmpliChip or activity score prediction models. xN: multiple copies of the allele detected; —, alleles not identified by platform.

#### XL-PCR+Sequencing

*CYP2D6* re-sequencing not only contributed to a comprehensive assessment of known *CYP2D6* sequence variations, but also allowed identification of novel allelic variants. A total of 92 sequence variations were identified including 88 SNPs, two insertions and two deletions (Additional file 5: Table S5). Additional novel SNPs were identified, but were not assigned to alleles as no apparent clinical relevance was observed (Additional file 5: Table S5). None of these SNPs were found to impact splicing based on the NetGene2 prediction.

Twenty one distinct alleles were identified in Cohort 2 (Additional file 6: Figure S1). Of the clinically relevant alleles identified by this platform, CYP2D6*17 and *5 were frequently observed in the Black population. In contrast CYP2D6*4 and *41 alleles were frequent in Caucasians and CYP2D6*4 in the Coloured population groups. Of the alleles identified by gene re-sequencing (n=200 successful identifications), 17.0% (n=34) had absent, 31.5% (n=63) decreased, 51.% (n=102) normal and 0.5% (n=1) increased enzyme function in the sampled cohort. All alleles described by sequencing were in Hardy-Weinberg Equilibrium.

#### Allele comparison between platforms

The most noticeable discrepancy between the two platforms was that AmpliChip identified fewer CYP2D6*2 alleles (not significant; *P*=0.107) and more *41 alleles (*P*<0.001). The normal function CYP2D6*45B (n=8) and *46 (n=1) alleles was not identified by AmpliChip and was incorrectly assigned as reduced function *41. Similarly, AmpliChip identified the non-functional CYP2D6*56B (n=1) as a reduced function CYP2D6*10B allele. Of the 200 alleles tested, AmpliChip identified nine CYP2D6*5 alleles compared to the seventeen identified by the Hersberger et al. [22] assay, of which only five subjects were accurately identified by both assays. This difference was significant (*P*=0.037). When investigated further, the Hersberger et al. [22] assay predicted nine individuals to be heterozygous CYP2D6*5 while AmpliChip reported homozygous *1 (n=1), *2 (n=1), *4 (n=1),*17 (n=5) or *41 (n=1) genotypes. Eighteen alleles identified by the XL-PCR+Sequencing platform were not identified by AmpliChip. In addition to CYP2D6*45B, *46 and *56B mentioned above, CYP2D6*59 (reduced function) was misidentified as CYP2D6*2 or *22 (both normal function). CYP2D6*33 and*43 (both normal function) were identified as CYP2D6*1 (normal function).

#### Predicted phenotype

There were more PMs identified in Cohort 1 than in Cohort 2. The XL-PCR+Sequencing platform did not identify more PMs in Cohort 2, but increased prediction of IMs. XL-PCR+Sequencing compared well with the cohort described by Wright et al. [17]; there were however, fewer PMs.

### *CYP2D6* novel alleles

**Figure 1:**
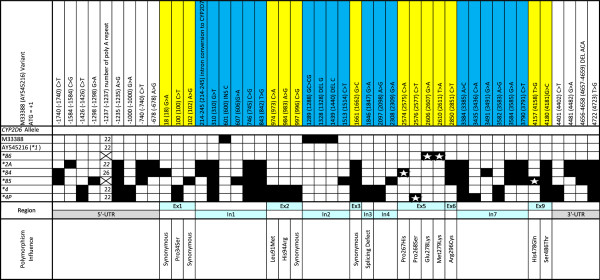
Schematic diagram representing novel CYP2D6*4P, *84, *85 and *86 alleles identified in the South African cohort described in this study. Polymorphisms are represented by black blocks. Allele defining polymorphism are indicated by a white star. These alleles have been accepted by the *CYP *Allele Nomenclature Committee for *CYP2D6 *(http://www.imm.ki.se/CYPalleles/). CYP2D6*2A and *4 used for comparison, were adopted from Gaedigk et al. [23]. BL: Black African; CA: Caucasian; IN: Indian; UTR: untranslated region; Ex: exon; In: intron.

Figure 1 displays the four novel alleles we identified by XL-PCR+Sequencing in comparison to other similar alleles. The CYP2D6*4P allele was found in a Caucasian individual. Due to the detrimental 1846 G>A SNP that causes aberrant splicing, CYP2D6*4P the novel 4157 T>G SNP did not require further characterisation. This allele received an AS of 0.0 classifying it as non-functional.

CYP2D6*84 has a *2A backbone and was found in a Black African individual. The allele-defining SNP 2574C>A in exon 5 that results in an amino acid change (P267H) was predicted to be benign by PolyPhen (PSIC score of 0.871), but SIFT predicted it to affect protein function (SIFT score of 0.03). The amino acid change was from a non-polar proline to a basic histidine, which results in a charge change, thereby supporting the possibility of altered activity. As there is a discrepancy between the *in silico* prediction tools, an AS score of 1.0 was given to this allele for comparative purposes.

CYP2D6*85 was also found in a Black African individual. The allele defining SNP for CYP2D6*85 was 4157 T>G that results in a H478Q amino acid change. According to PolyPhen (PSIC=0.419) and SIFT (SIFT= 0.58) this change is unlikely to affect activity. Therefore, we assigned an AS of 1.0 to this allele. CYP2D6*85 also has a CYP2D6*2 backbone.

The final novel allele, CYP2D6*86, was discovered in an Indian individual. Only two SNPs 2606 G>A and 2610 T>A were observed, and both caused an amino acid change, i.e. E278K and M279K. However, only 2610 T>A was predicted to be likely to affect protein function by PolyPhen, (PSIC=1.905, SIFT=0.01) due to a hydrophobicity change from a non-polar to a basic amino acid in a buried site. The other SNP, 2606 G>A, is unlikely to affect enzyme activity (PSIC=0.205, SIFT=0.07). However, because the 2610 T>A was not confirmed to alter activity, the CYP2D6*86 allele was assigned an AS score of 1.0 for comparative purposes. Both SNPs have been described previously, but not within a defined allele (Tandon et al. manuscript in preparation, http://www.cypalleles.ki.se/cyp2d6.htm).

## Discussion

The ability of AmpliChip to simultaneously assay for *CYP2D6* gene duplications, gene deletions as well as 33 *CYP2D6* and 3 *CYP2C19* variants simultaneously, characterises it as high-throughput. However, several limitations were identified which question the use of AmpliChip in the South African population.

First, AmpliChip performed poorly in terms of reliability. For *CYP2D6* the average failure rate in both groups was 22.4%. In addition, only 2 out of the 13 samples that failed on first attempt succeeded after a second attempt, raising the concern of cost effectiveness. Possible explanations for the poor success rate of AmpliChip in this population include (i) suboptimal transportation conditions and mishandling during transfer possibly damaging the microarrays; (ii) concerns regarding the length of the amplification - this has previously been suggested to be a weak point in the assay [7]. Rebsamen et al. [9] supported this proposal and identified gene duplication errors. However, each amplification reaction was tested for product using 1.0% agarose gel electrophoresis prior to proceeding to the fragmentation step. The failures observed are therefore unlikely to be due to the lack of a PCR product; (iii) inadequate fragmentation, which in turn impacts on hybridisation, thereby rendering the test a failure. In 2007, the FDA reported that the DNase I recommended in the AmpliChip information leaflet was of reduced quality, resulting in low specific activity (http://www.fda.gov/Safety/Recalls/EnforcementReports/2007/ucm120450.htm); (iv) lack of standardisation of the strepravidin R-phycoerythrin conjugate. Roche has stopped supplying the recommended reagent and has not recommended a suitable replacement.

The high frequency of unknown predicted phenotypes called by AmpliChip is a serious limitation for routine implementation in the South African population. Approximately 7.7% predicted phenotypes were “Unknown” even though AmpliChip was successful. These individuals would not have benefited from pharmacogenetic screening by AmpliChip for *CYP2D6*. This questions the use of this pharmacogenetic screening assay in the South African population as the frequency of the “Unknown” predicted phenotype is higher than the frequency of PMs identified (1.0-9.6%). With AmpliChip being more expensive that the other platforms and having a low success rate, AmpliChip will not assist in reducing the financial burden associated with CYP2C19 and CYP2D6 associated ADRs.

### AmpliChip compared to *CYP2C19* PCR-RFLP platform

The ability to cover population-specific alleles is another limitation. AmpliChip may have had a high success rate for *CYP2C19*, and the frequencies compared well with previously reported values in various African populations [7,35], but there may be several mutations which AmpliChip was unable to identify (i.e. *CYP2C19* alleles *2 and *3 were discovered). Thus, there may be a higher frequency of alternative polymorphisms resulting in absent (CYP2C19*4, *5, *6, *7 and *8) or increased (*17) enzyme function (http://www.cypalleles.ki.se/). Additionally, a glimpse into the South African Xhosa and Cape Mixed Ancestry (Coloured) populations has revealed a novel mutation in the promoter region -1041 G>A (CYP2C19*27) which was found to be present at a relatively high frequency of 33.0% [18]. *In silico* analyses and luciferase expression assays suggest that this polymorphism may result in reduced expression of *CYP2C19*[18]. The absence of these important alleles from AmpliChip highlights the need to develop a more specific and/or comprehensive assay for this population.

The more comprehensive PCR-RFLP genotyping method identified 83 alleles out of 158 that were wrongly assigned by AmpliChip as “CYP2C19*1” (i.e. wild type and unidentified alleles). This is significant for the accuracy of downstream phenotype prediction and agrees with concerns that the *CYP2C19* alleles identified by AmpliChip, would not be comprehensive enough for the South African population. The incorrect assignment of “CYP2C19*1” was especially relevant to the Black South African cohort, as 48.6% of the alleles initially assigned as “CYP2C19*1” by AmpliChip, were assigned other alleles after PCR-RFLP genotyping (Table 3). However, the effect of these alleles needs to be carefully considered before drawing firm conclusions.

The variation in the LD pattern observed for the CYP2C19*2 and *27 defining SNPs, identified in the three Black Africans and one Indian individual, but was not observed in the small Caucasian cohort. This alternative LD was identified previously in a Black African population [18] and one should be aware of the clinical implications of this. For example, if CYP2C19*27 was responsible for decreased metabolism, an individual testing positive for both the *2 and *27 alleles could be *2/*27 (AS=0.5) or *2+*27/*1 (AS=1). The low LD observed predominantly in Africans may complicate the assignment of alleles and may necessitate the genotyping of multiple SNPs before allele assignment.

Considering the high frequencies observed for CYP2C19*17 in a variety of populations [36-40] and the identification of other high frequency alleles such as *27[18], which may have clinical implications, it could be argued that AmpliChip is not comprehensive enough for any population. In addition, AmpliChip is a relatively expensive assay for prediction of CYP2C19 phenotype and a population specific, reasonably priced assay such as PCR-RFLP is advised for future phenotype prediction, especially in developing countries where resources are limited.

### AmpliChip compared to the *CYP2D6* XL-PCR+Sequencing platform

As our cohort represented a diverse population it was not surprising to find a large number of *CYP2D6* allelic variants as well as four novel alleles. Nine CYP2D6*2 alleles were miss-called as *41, resulting in an over estimation of CYP2D6*41/*41 homozygotes [41]. The AmpliChip-derived frequency of CYP2D6*41 among our Black subjects was therefore higher when compared to similar cohorts [35], in which the CYP2D6*41 allele was detected by its key SNP (2988 G>A). AmpliChip designates CYP2D6*41 using the -1584 C>G variation and linkage disequilibrium with other SNPs, which generally hold true in Caucasians, but not in subjects of Black African ancestry [41]. Furthermore, using the CYP2D6*41 key SNP will also allow differentiation of CYP2D6*41 from *45B and *46 alleles which are not identified by AmpliChip.

CYP2D6*1 was incorrectly assigned as *41 by AmpliChip five times. This could possibly be due to the lack of hybridisation. These inaccurate genotype assignments affect the prediction of subjects’ phenotypes to various extents. In addition, AmpliChip does not contain identifying, or key SNPs for CYP2D6*45, *46, *56 and *59, which we have discovered by the XL-PCR+Sequencing platform and hence, defaulted these alleles to CYP2D6*2 or *10 according to the AmpliChip algorithm. Inaccurate results in combination with alleles that are not captured by AmpliChip could have serious pharmacogenetic and clinical implications.

### Predicted phenotype

There was a noticeable difference in phenotypic prediction between AmpliChip and the AS for both CYP2D6 and CYP2C19. This was apparent when comparing each system on both a group to group and combined level. Accurate phenotype prediction appears to be a limitation of AmpliChip which supports the use of a numeric method for phenotype identification [7,9,30]. In addition, a 93% CYP2C19 EM prediction may be an overestimate. Use of the numeric AS allows for CYP2C19 IM to be predicted; this is a subset of the cohort that could potentially benefit from pharmacogenetic screening. Articles comparing clopidogrel (prodrug) response to *CYP2C19* variability have demonstrated reduced metabolism in individuals who have CYP2C19*1/*2 or *1/*3 allele combinations. These genotypes were associated with normal or only slightly reduced platelet aggregation, as clopidogrel needs to be metabolised into its active metabolite in order to affect platelet aggregation [42-44]. It may therefore be more appropriate to split this EM group into EM and IM. In this way *1/*2 and *1/*3 individuals could potentially benefit from pharmacogenetic screening. Measured phenotype would be needed to fully understand and evaluate phenotype prediction by the various platforms. With the AmpliChip not identifying the increased function CYP2C19*17, tailoring of clopidogrel dosage would be difficult.

### Pharmacogenetic relevance for the South African population

The possible existence of additional functionally relevant alleles unique to the South African population will need to be considered if *CYP2D6* and *CYP2C19* pharmacogenetics are to be applied in this population [45]. With the large amount of genetic variation observed in this South African cohort it would be essential to use more comprehensive platforms for pharmacogenetic screening to ensure a more accurate predicted phenotype. Predicted phenotype may not be clinically relevant if genotyping is incomplete and inaccurate, highlighting the importance of establishing novel approaches for predicting phenotype [7,30]. It will also be important to compare genotype and measured phenotype in this population, to assess the accuracy of the predicted phenotype called by AmpliChip [14] as well as other prediction strategies. Due to the high failure rate and high cost of AmpliChip it is not feasible to repeat these AmpliChips to evaluate the cause for error.

## Conclusion

When applied to a demographically-representative sample of the South African population, the AmpliChip had a low success rate and a high number of unknown predicted phenotype calls were observed. This platform would need to be refined before being applied as a pre-prescription pharmacogenetic screening tool in this, and possibly other genetically-diverse African populations. Alternative platforms for genotyping, such as the ones used in this study, would be more clinically appropriate for pharmacogenetic screening of *CYP2D6* and *CYP2C19*. With the rapid advance in sequencing technologies, read lengths are improving which will allow sequencing to form the basis of pharmacogenetics in the future. This will facilitate simultaneous identification of novel alleles in complex populations. The comparison between predicted phenotype and measured phenotype will also need to be considered.

## Competing interests

The authors would like to declare that they have no competing interests.

## Authors’ contributions

TMD along with HF carried out the molecular evaluation of Cohort 2 using the AmpliChip CYP450 Test. TMD wrote this manuscript, assisted with the recruitment of Group 1, assisted in securing some funding, was involved in all Cohort 2 genotyping and performed all statistical analyses. WEH and SMA recruited and carried out the molecular evaluation of Cohort 1. CD supervised work carried out with Group 2, assisted in securing funding for this group and contributed to study design. PR assisted in sampling Group 2 and contributed to study design. BID, GEBW and LW assisted with *CYP2C19* genotyping. CDJL and AVS assisted in the sequencing of *CYP2D6*. AG assisted with various concepts related to *CYP2D6* analysis. MSP designed the study, oversaw most experiments and secured the majority of funding needed. All authors have read and approved the final manuscript.

## Pre-publication history

The pre-publication history for this paper can be accessed here:

http://www.biomedcentral.com/1471-2350/14/20/prepub

## Supplementary Material

Additional file 1: Table S1 PCR specifications for amplification of the region of interest specific to each *CYP2C19 *allele (Drögemöller *et al*. 2010). Click here for file

Additional file 2: Table S2 RFLP specifications for specific *CYP2C19 *allele identification (Drögemöller *et al*. 2010). Click here for file

Additional file 3: Table S3 PCR specifications for *CYP2D6 *amplification and characterisation. Click here for file

Additional file 4: Table S4 Primers used to detect and describe polymorphisms associated with *CYP2D6* alleles, ordered according to gene orientation 5' to 3'. Click here for file

Additional file 5: Table S5 *CYP2D6 *polymorphisms identified in a demographically representative South African cohort. Click here for file

Additional file 6: Figure S1. Schematic diagram demonstrating *CYP2D6 *SNPs associated with variants identified in a demographic South African cohort. Click here for file
